# Exploring scale-up, spread, and sustainability: an instrumental case study tracing an innovation to enhance dysphagia care

**DOI:** 10.1186/1748-5908-8-128

**Published:** 2013-10-29

**Authors:** Irene Ilott, Kate Gerrish, Sue Pownall, Sabrina Eltringham, Andrew Booth

**Affiliations:** 1 NIHR CLAHRC-SY, Sheffield Teaching Hospitals NHS Foundation Trust, 11 Broomfield Road, Sheffield S10 2SE, England; 2 School of Nursing and Midwifery, University of Sheffield/ Sheffield Teaching Hospitals, NHS Foundation Trust, 11 Broomfield Road, Sheffield S10 2SE, England; 3 Sheffield Teaching Hospitals NHS Foundation Trust, 4 Claremont Crescent, Sheffield S10 2JF, England; 4 Resources Group, HEDS, ScHARR, The University of Sheffield, Regent Court, 30 Regent Street, Sheffield S1 4D, England

**Keywords:** Dysphagia, Spread, Sustainability, Stroke, Fractured neck of femur

## Abstract

**Background:**

Adoption, adaptation, scale-up, spread, and sustainability are ill-defined, undertheorised, and little-researched implementation science concepts. An instrumental case study will track the adoption and adaptation, or not, of a locally developed innovation about dysphagia as a patient safety issue. The case study will examine a conceptual framework with a continuum of spread comprising hierarchical control or ‘making it happen’, participatory adaptation or ‘help it happen’, and facilitated evolution or ‘let it happen’.

**Methods:**

This case study is a prospective, longitudinal design using mixed methods. The fifteen-month (October 2012 to December 2013) instrumental case study is set in large, healthcare organisation in England. The innovation refers to introducing a nationally recognised, inter-disciplinary dysphagia competency framework to guide workforce development about fundamental aspects of care. Adoption and adaptation will be examined at an organisational level and along two, contrasting care pathways: stroke and fractured neck of femur. A number of educational interventions will be deployed, including training a cadre of trainers to cascade the essentials of dysphagia management and developing a Dysphagia Toolkit as a learning resource. Mixed methods will be used to investigate scale-up, spread, and sustainability in acute and community settings. A purposive sample of senior managers and clinical leaders will be interviewed to identify path dependency or the context specific particularities of implementation. A pre- and post-evaluation, using mealtime observations and a survey, will investigate the learning effect on staff adherence to patient specific dysphagia recommendations and attitudes towards dysphagia, respectively. Official documents and an ethnographic field journal allow critical junctures, temporal aspects and confounding factors to be explored.

**Discussion:**

Researching spread and sustainability presents methodological and practical challenges. These include fidelity, adaptation latitude, time, and organisational changes. An instrumental case study will allow these confounding factors to be tracked over time and in place. The case study is underpinned by, and will test a conceptual framework about spread, to explore theoretical generalizability.

## Background

There is increasing concern about the slow, haphazard way in which healthcare organisations implement and then sustain clinically and cost effective innovations. In England, the National Health Service (NHS) Institute for Innovation and Improvement [1] estimate that up to 70% of all organisational change does not survive. This is a waste of time, financial resources, and leadership effort at a time of economic austerity. Implementation, whether from research or other sources of knowledge, is much more than the simple adoption and diffusion of innovations [2].

Adoption, adaptation, scale-up, spread, and sustainability are ill-defined, under-theorised, and little-researched concepts [3-6]. The following definitions illustrate some subtle differences between these terms. The concept of adoption draws on Everett Roger’s classic Diffusion of Innovations Theory [7]. Rogers offers a five-stage decision-making adoption process that comprises awareness, interest, evaluation, trial, and adoption. Adaptation refers to tailoring the innovation and/or intervention to the local conditions. Ovretveit suggests that adaptation latitude, or the freedom to modify the innovation to fit the setting, is important for ownership [5]. Scale-up or large-scale improvement [8] are often used in the context of international, national and regional health programmes. These terms refer to deliberate, systematic approaches to increasing the coverage, range, and sustainability of services [9]. Scale-up may be seen as the equivalent of vertical diffusion as it is planned strategically, in a top-down way. In contrast, spread is associated with horizontal diffusion, for example, with an innovation spreading along a care pathway. The Institute for Healthcare Improvement describe spread in ambitious, if not aspirational terms, as ‘actively disseminating best practice and knowledge … and implementing each intervention in every available care setting’ and sustainability as ‘locking in the progress … and continually building upon it’ [10], p.3. Finally, Buchanan *et al.* define sustainability in healthcare modernization as ‘the process through which new working methods, performance enhancements, and continuous improvements are maintained for a period appropriate to a given context’ [3], p.231. They contrast sustainability with decay, where change is not maintained and the benefits are lost.

This protocol describes an instrumental case study designed to examine the spread and sustainability of a locally developed healthcare innovation. We present the background to the innovation—a recommendation about considering dysphagia as a patient safety issue that required an organisation wide approach to staff development.

### The research opportunity

In 2009, enhancing dysphagia management was selected as a healthcare service priority for a collaborative knowledge translation project to be undertaken by the National Institute for Health Research (NIHR) Collaboration for Leadership in Applied Health Research and Care for South Yorkshire (CLAHRC SY). For further information see, http://clahrc-sy.nihr.ac.uk/.

Dysphagia is a serious problem because swallowing problems compromise health and quality of life. Swallowing problems increase the risk of malnutrition, dehydration, aspiration, and pneumonia, and can lead to death [11,12]. Dysphagia is very distressing, for both the individual and their family, jeopardising social participation. The ‘severe’ negative social and psychological impact of dysphagia was highlighted in a qualitative study with 360 participants from four European countries with participants attributing their ‘increased sense of isolation and loss of self-esteem to swallowing problems’ [13], p.143.

The twelve-month feasibility study evaluated workplace based, blended e-learning about dysphagia on a stroke rehabilitation ward [14]. A single group, pre-post design was employed to examine change in attitude, knowledge, and practice of the population of registered nurses and health care assistants. Blended e-learning, the knowledge translation strategy, involved study sessions with a needs analysis, the e-learning programmes, practical skills training about modifying liquids according to national descriptors, and action planning for putting new learning into practice. The learning effect was evident on the post and follow-up measures, with some items of dysphagia knowledge and attitude achieving significance at the p ≤0.05 level. The most common self-reported changes in practice related to medicines management, thickening fluids and oral hygiene. All participants achieved a nationally recognised level of competence as Assistant Dysphagia Practitioners [15].

The recommendations arising from the knowledge translation project promoted dysphagia as vital for patient safety. The research will track one specific recommendation about using the Inter-Professional Dysphagia Competence Framework [15] for workforce development. This is described as an innovation because, as far as we are aware, this is the first time that the Framework has underpinned a strategy for workforce development at scale in a public health care organisation. It is a novel approach that is intended to improve health outcomes and will be implemented as a planned change [16]. The Inter-Professional Dysphagia Competence Framework [15] was developed to offer a consistent approach to education in the United Kingdom. It is recognised by relevant professional associations including the Royal College of Nursing; the Royal College of Physicians; the Royal College of Speech and Language Therapists and the British Dietetics Association. The Framework comprises five levels. Firstly, there is awareness, which introduces the risks of dysphagia. The second level is Assistant Dysphagia Practitioner, which identifies the knowledge and skills for patient safety and applies to anyone who assists patients to eat or drink. Next, is Foundation Dysphagia Practitioner competence, which covers protocol-guided assessment of swallowing. The fourth level is Specialist Dysphagia Practitioner competence to assess and manage dysphagia. Finally, Consultant Dysphagia Practitioners are competent to undertake specialist investigations, manage complex cases, and contribute to research.

### Conceptual framework

A recent review about sustainability recommends that future research should use a conceptual framework about sustainability, characterise the innovation, and study the factors that influence changes in implementation over time [6]. This research will address these points. First, a conceptual framework about spread derived from the international healthcare literature [5] underpins the study. Second, the characteristics of the innovation are explicit in the dysphagia recommendations. Finally, sustainability will be investigated prospectively in a specific context.

An existing conceptual framework to spread [5] underpins the study. This framework proposes three approaches to spread—hierarchical control, participatory adaptation, and facilitated evolution. Hierarchical control refers to a top-down ‘push’ with senior-level decision making and operational staff held to account for making the change. Participatory adaptation is where decision making is more participatory, focusing on principles and examples; and support is provided for local adaptation alongside regular feedback from experts. Facilitated evolution aims to create the conditions for self-initiated change in response to need. The emphasis is on capacity building, facilitation, making resources available to solve the problem, rather than prescribing change and the adoption process. These approaches reflect the continuum of spread in service organisations noted by Bate *et al.*[17] who refer to ‘make it happen’, ‘help it happen’, and ‘let it happen’. The research will explore whether this descriptive framework aids understanding of the implementation of the dysphagia recommendations.

The research opportunity provides a natural vehicle to study some relatively neglected aspects of implementation, by applying a conceptual framework about spread. The research aims are to:

1. Examine the processes and outcomes associated with the diffusion of a locally developed innovation about dysphagia.

2. Identify the factors that influence the adoption, adaptation, scale-up, spread, and sustainability of the dysphagia innovation.

3. Develop guidance to support longer-term change in a healthcare organization.

## Methods

### Setting

The research is set in a public healthcare organisation in the middle of England that employs around 15,000 staff and provides care in hospitals, community facilities and peoples’ homes. Critically, the internal context—the healthcare organisation—is receptive towards the research for a number of reasons. First, dysphagia was identified as a priority for a CLAHRC SY project by the Executive Lead Nurse in 2009. Next, the study builds on the success in the Stroke Service were a local innovation—workplace based, blended e-learning—was used to deliver job specific training which had an impact on knowledge, attitudes, and practice. Both these reasons are likely to contribute to psychological ‘buy-in’ to the spread and sustainability of the recommendations. Third, in October 2011, the organisation established mechanisms to respond to the recommendations about dysphagia as a patient safety issue and there is a strong commitment to supporting scale-up. Finally, the research team are embedded within the organisation, working in partnership with key decision makers and clinicians. This context provides a natural opportunity for an instrumental case study in a receptive organisation.

### Design

The study is an instrumental case study, using a prospective, longitudinal design and employing mixed methods. In an instrumental case study, a specific instance is examined to understand a general principle [18]. The implementation of the dysphagia recommendation is being studied to learn about spread and sustainability. The dysphagia recommendation will be viewed as a tracer innovation, which means that adoption and adaptation will be tracked for fifteen months (October 2012 to December 2013) after the recommendations were disseminated within the organisation in April 2011. The research involves prospective tracking at clinical and organisational levels. The clinical level refers to the care pathways for stroke and fractured neck of femur. The organisation is a publically funded healthcare organisation.

At the clinical level, we will train a cadre of local trainers on two care pathways to facilitate spread, as participatory adaptation, emphasizing collaborative support for the adoption and local adaptation. The trainers will be equipped to deliver the first two levels on the Inter-Professional Dysphagia Competence Framework [15], namely Awareness and Assistant Dysphagia Practitioner competence. The care pathways relate to stroke and fractured neck of femur, both of which integrate acute services with rehabilitation in community facilities. We will be exploring the spread of the dysphagia recommendations to the care pathway for fractured neck of femur, and the spread and sustainability in the stroke care pathway.

These two care pathways were chosen because they complement each other on a number of dimensions. Dysphagia is a common, expected problem post-stroke, whereas it is less so for frail, older people who have fallen and fractured their femur. The incidence of dysphagia post-stroke ranges from 37% to 78% [19]. Dysphagia is a serious risk factor, albeit more indirectly following a hip fracture. A recent meta-analysis estimated the prevalence of dementia amongst older hip fracture patients at 19% and the prevalence of cognitive impairment was estimated at 42% [20]. Dysphagia is a common symptom in dementia. It has been estimated that up to 45% of patients institutionalized with dementia have some degree of swallowing difficulty [21]. Another dimension is the profile of dysphagia in cognate guidelines. The National Clinical Guideline for Stroke states that patients with acute stroke should have their swallowing screened within four hours of admission [22], p.58. Although fluid and nutritional status is part of the NHS Institute for Innovation and Improvement [23] care pathway for fractured neck of femur, nutrition is mentioned in a single case study in the National Hip Fracture Database National Report [24].

At the organisational level, implementation will be tracked through formalisation of the dysphagia recommendation in organisation-wide staff education policies and procedures, reflecting hierarchical control. Also, a Dysphagia Toolkit—a set of resources to support the essentials of dysphagia management—will be put on the Trust intranet and promoted using the mantra ‘patient safety: dysphagia matters’. This reflects facilitated evolution whereby resources are made available to meet anticipated demand, so that ‘take-up sites’ are able to find, adapt, and tailor the innovation as a solution to their problem.

At both levels, we will ‘adopt a processual stance, focusing on the substance and process of change in context’ [25], p.202. The dysphagia recommendation about workforce development is the substance. The process encompasses adopting and adapting this recommendation. This processual stance assumes a path dependency [3] with implementation being dependent upon prior events. Temporal factors, including the timing, sequencing, and pacing of events will be taken into account as they may explain the outcomes. Critical junctures or factors triggering action, the decisions of key stakeholders over time, and the patterns of positive and negative responses to these decisions, will be explored. The context is the organisational setting and external factors that may assist or impede spread and sustainability. According to Greenhalgh *et al.* “context’ and ‘confounders’ lie at the very heart of dissemination, implementation and sustainability. They are not extraneous to the object of study—they are an integral part of it. The multiple (and often unpredictable) interactions that arise in particular contexts and settings are precisely what determine the success or failure of the spread/sustainability initiative’ [16], p.322. Such a focus on the processes of change in context is intended to capture the ‘cumulative effects’ and ‘conjunctural causality’ where a build-up of pressures or a combination of factors support the desired outcomes [3].

### Interventions

Most interventions relate to education. ‘Train-the-trainer’ and ‘develop materials’ feature within a recent review [26] that identifies six key processes and defines 68 implementation strategies. A cadre of trainers will be prepared and supported to deliver the dysphagia Awareness and Assistant Dysphagia Practitioner competence-level training to relevant staff in each care pathway. This involves other strategies including ‘educate through peers’, ‘distribute educational materials’, ‘conduct on-going training’, and ‘educational outreach visits’. The Dysphagia Toolkit, which contains teaching resources for the trainers and about dsyphagia, represents ‘develop materials’ and ‘increase demand’ for further training.

### Participants

The participants will be a purposive sample of senior managers with an organisation-wide remit and front-line staff from the two care pathways. Senior managers (n = 6 to 8) include relevant departmental managers, including Learning and Development, Speech and Language Therapy and senior nurses. The stroke and fractured neck of femur care pathways comprise five hospital wards and two community facilities offering immediate post-operative care, acute care and rehabilitation. The three groups of front-line staff include those who assist patients to eat and drink (n = 200 to 250), for example, healthcare assistants, registered nurses, and ward housekeepers; the nurse leaders on the wards (n = 7); staff responsible for clinical education and those who participated in the ‘train the trainer’ intervention (n = 8 to 10).

### Data collection

Case study data will be gathered through multiple methods, triangulated and then interpreted using the conceptual framework outlined above. The methods are semi-structured interviews, a questionnaire survey, structured observation of staff behaviour at mealtimes and documentary analysis. Given the empirical challenge of evaluating sustainability and spread [6] we will reflect on the utility of the design.

Post-intervention, semi-structured interviews will be held with senior managers, clinical leaders, and educators. The objectives will be to examine the influence of priorities, perceptions, and relationships on the implementation of the dysphagia recommendations; and to investigate the expected and unexpected ways in which the recommendations were adopted, adapted, sustained, and spread in different settings over time. The semi-structured interviews with senior managers will explore barriers and facilitators to implementation and perceptions about the effectiveness of the three approaches. The post-intervention interviews with clinical leaders and educators will consider the training the trainer intervention as participatory adaptation, and elicit changes in practice.

A brief questionnaire will be distributed to all front-line staff who assist patients to eat and drink on the two clinical pathways, before and after the ‘training the trainers’ intervention. The objective is to identify the learning effect, particularly whether there are any differences in attitude towards dysphagia. This will be assessed using a validated measure, the 21-item Mealtime and Dysphagia Questionnaire [27] that examines three barriers to adherence. These are: hassle, the difficulty and extra work required; lack of knowledge of feeding techniques; and disagreement with treatment recommendations. Demographic information and details about dysphagia training will also be collected.

At least three mealtime observations will be carried out before and after the ‘training the trainers’ intervention in each of the seven clinical areas that form the care pathways. The objectives are: to gain an insight into current dysphagia management; to compare adherence to dysphagia management recommendations before and after the ‘training the trainers’ intervention; and to identify any change in practice. A structured observation form will be used to investigate whether the recommendations are being put into practice by staff to enhance the care of patients with dysphagia. A bespoke, rather than existing observation tool will be used for two reasons. First, most assessment tools measure patients’ performance and few are psychometrically robust [28]. Second, we are interested in staff adherence to patient specific, dysphagia recommendations. All the observations will be done as unobtrusively as possible to minimise the distraction at mealtimes and to reduce the reactive effect on staff behaviour [29]. The observations will be done by a speech and language therapist, who will intervene if, in their judgement, patient safety is compromised.

The documentary analysis will draw on two data sources to trace the substance and process of change in context. First, organisation wide policies and procedures will be used to track the adoption and adaption of the dysphagia recommendations. Second, a contemporaneous, ethnographic field work journal [30] started in April 2011 records the particularities of the case and each approach, including flow of events, critical junctures, activities, key agents, and outcomes. Official documents are likely to indicate the formalisation of the dysphagia recommendation; for example, whether the Inter-Professional Dysphagia Framework for workforce development has been introduced into local induction programmes. The researchers will summarise events and behaviour, note their initial reflections and record any organisational changes in the fieldwork journal. The primary aim is to assist recall about dissemination, to offer insights into the processes of spread and sustainability, and to inform analysis and interpretation.

### Ethics

Ethical approval for this study has been granted by the University of Sheffield Ethics Committee (Reference number ERP 125; approval granted 18/07/2012). Informed consent will be sought from all participants, including having the interviews recorded and transcribed. All identifying information about the organisation will be removed from the documents and fieldwork journal. All identifying information about participants from interviews and survey completion will be removed. Data analysis will consist of collated information only. All research data will be anonymized and stored electronically (password protected) on the Sheffield Teaching Hospitals NHS Foundation Trust network.

### Data analysis

The processes and outcomes will be explored to identify success factors that may be generalisable to other healthcare innovations and settings. In addition, the utility of the conceptual framework will be reviewed and the lessons learned will be summarised in a briefing paper for the CLAHRC SY website.

All interviews will be recorded with the participant’s permission, transcribed verbatim and analysed using the five-stage Framework Approach [31]. The stages are: familiarization, gained through listening and reading the transcripts; identifying the thematic framework, this will be developed deductively, with the conceptual frameworks referred to earlier, informing the themes; indexing and coding the data using NVivo (QSR NVivo version 8), specialist software, independently by two researchers; charting or interrogating each coding category in the thematic framework; and mapping and interpretation which involves drawing the dataset back together as a whole to explore patterns and associations.

Any patterns observed during the structured observations will be identified using descriptive statistics and the Framework Approach. Staff adherence, behaviour and skills will be summed and synthesized with the notes about the physical and social environment at mealtimes.

Each care pathway will serve as the unit of analysis. Non-parametric tests will be used to compare groups and examine any difference in staff attitudes. The fieldwork journal will be cross checked to trace and verify the timeline, and to explore the path dependency and processes associated with the organisational- and clinical-level interventions. The pre-post design will be appraised, to highlight contextual, temporal, and confounding factors, particularly the linkages between, and the cumulative effect of the three approaches to spread.

Finally, the data from the different methods will be triangulated. Trends and differences will be explored to identify influences and success factors associated each approach. Our interpretation of the findings and conceptual framework will be discussed with the research advisory team, which includes patient and public members, to check credibility and to minimise the risk of bias, including observer bias.

The researchers’ role as boundary spanners will be taken into account as another source of bias. Their role is to help the innovation to spread through negotiation, influencing, and enablement across organisational and professional boundaries, and then evaluate the processes and outcomes. The research team straddle organisational boundaries being employed by the healthcare organisation, which hosts the CLAHRC SY. As such, they combine insider-outsider views, which brings benefits in terms of close working relationships but also risks of bias, due to their commitment to enhancing the quality of care for people with dysphagia.

## Discussion

An instrumental case study will explore scale-up, spread, and sustainability. These are essential, yet relatively neglected aspects of implementation. This is unsurprising for a variety of reasons. These include the tendency for innovations to decay [1]. The concepts are ambiguous and used synonymously, something that we have done too. Many factors unconnected to the innovation may impede spread and sustainability. For example, the lack of a national target in England about dysphagia, and therefore associated financial incentives, may add to the implementation challenge for this local innovation. Sixteen million pounds or 2.5% of the organisation’s income for 2012 and 2013 is dependent on the achievement of national quality targets. Some national quality standards in the United Kingdom refer to dysphagia, and there is one specific target for stroke. Outcome 5C of the Care Quality Commission standards [32] requires providers to identify people with swallowing difficulties and to take appropriate action. The target is a Stroke Quality Standard about patients with acute stroke having their swallowing screened by a specially trained healthcare professional within four hours of admission to hospital [33]. However, presenting dysphagia as a patient safety issue is a compelling narrative that has, to date, secured inter-professional engagement from all levels of the organisation. This has overcome a key improvement challenge—convincing people that there is a problem [34].

There are methodological problems, particularly in relation to time, adaptation, and fidelity [6]. New services change and develop over time. The prospective design means that spread and sustainability will be studied in a natural setting in real time. The ongoing tension between implementation fidelity and adaptation latitude supports the notion of an innovation with core components and an adaptable periphery [35]. In this research, the core has a broad purpose and mechanism. The purpose is to promote dysphagia as a patient safety issue and the mechanism is the organisation adopting the Interdisciplinary Dysphagia Competence Framework for workforce development. This means that local adaptation is legitimate and encouraged.

The researchers’ role as boundary spanners is both a strength and a weakness. Boundary spanners are said to be the human force behind knowledge transfer [36] because they connect the worlds of research and practice. The essence of boundary spanning is ‘getting the right mix of people and information together to tackle the right issue at the right time’ [36], p.9. This definition corresponds with the collaborative, co-production principles that underpin the NIHR CLAHRC SY. However, it is also a source of bias due to the researchers’ commitment to considering dysphagia as a patient safety issue.

The conceptual framework will allow three approaches—hierarchical control, participatory adaptation, and facilitated evolution—derived from international health to be applied to a local innovation [5]. However, measuring the outcomes associated with each approach, and assigning attribution, will be problematical. The research will test the feasibility and utility of diverse indicators of impact. These include documentary evidence that the Dysphagia Competence Framework has been put into Trust policies (hierarchical control or ‘make it happen’); the number of staff trained by the trainers (participatory adaptation or ‘help it happen’); and the hit statistics for the Dysphagia Toolkit on the Trust intranet (facilitated evolution or ‘let it happen’).

Exploring staff perceptions is likely to reveal whether the conceptual framework about spread is helpful and transferrable. The overlaps, linkages and accumulative effects between the approaches will be considered when tracking the innovation across time and *in situ*, to explain the change process. We recognise that this processual-contextual perspective will limit the transferability of the findings. This is because the ‘cumulative effects’ and ‘conjunctural causality’ [3], where a build-up of pressures or a combination of factors support the desired outcomes, are likely to be context-specific. However, as Yin observes: ‘case studies are generalizable to theoretical propositions, not to populations or to universes … your goal will be expand and generalize theories (analytical generalizability)’ [37], p.15. We believe that the strategic choice of this dysphagia case, for all the reasons rehearsed above, will maximise the usefulness of the findings in advancing theory in this important, yet neglected area.

## Competing interests

The authors declare that they have no competing interests.

## Authors’ contributions

II, KG, and SP concieved the study. AB and SE participated in the design. All authors read and approved the final manuscript.
